# Women’s preferences for childbirth experiences in the Republic of Ireland; a mixed methods study

**DOI:** 10.1186/s12884-016-1196-1

**Published:** 2017-01-10

**Authors:** Patricia Larkin, Cecily M. Begley, Declan Devane

**Affiliations:** 1 School of Health and Science, Scoil na Sláinte agus na hEolaíochta, Dundalk Institute of Technology, Dublin Road, Dundalk, Co. Louth, Ireland; 2 School of Nursing and Midwifery, Trinity College Dublin, Dublin 2, Ireland; 3 School of Nursing and Midwifery, National University of Ireland Galway, Galway, Ireland

**Keywords:** Childbirth experiences, Mixed methods, Discrete Choice Experiment

## Abstract

**Background:**

How women experience childbirth is acknowledged as critical to the postnatal wellbeing of mother and baby. However there is a knowledge deficit in identifying the important elements of these experiences in order to enhance care. This study elicits women’s preferences for the most important elements of their childbirth experiences.

**Methods:**

A mixed methods design was used. An initial qualitative phase (reported previously) was followed by a second quantitative one using a discrete choice experiment (DCE), which is reported on here. Participants who had experienced labour, were over 18 and had a healthy baby were recruited from four randomly selected and one pilot hospital in the Republic of Ireland. Data were collected by means of a DCE survey instrument. Questions were piloted, refined, and then arranged in eight pair-wise scenarios. Women identified their preferences by choosing one scenario over another. Nine hundred and five women were sent the DCE three months after childbirth, with a response rate of 59.3% (*N* =531).

**Results:**

Women clearly identified priorities for their childbirth experiences as: the availability of pain relief, partnership with the midwife, and individualised care being the most important attributes. In the context of other choices, women rated decision-making, presence of a consultant, and interventions as less important elements. Comments from open questions provided contextual information about their choices.

**Conclusions:**

Most women did not want to be typified as wanting the dichotomy of ‘all natural’ or ‘all technology’ births but wanted ‘the best of both worlds’. The results suggest that availability of pain relief was the most important element of women’s childbirth experiences, and superseded all other elements including partnership with the midwife which was the second most important attribute. The preferences identified might reflect the busy medicalised hospital environments, in which the vast majority of women had given birth, and may differ in settings such as midwifery led care or home births.

## Background

A woman’s experience of labour and birth can have a profound impact on her wellbeing and that of her baby, partner, and family [1, 2]. The childbirth experience has been long recognised as a life-altering event. It has been found to have a powerful lasting potential to enhance or detract from women’s feelings of self-confidence and these feelings often last a lifetime [3, 4]. In Ireland, there is a dearth of research on women’s views about important elements of their childbirth experiences. There is little information therefore about whether the maternity services provided actually meet women’s needs. The increasing emphasis on choice and continuity for maternity services such as those developed in the UK and Northern Ireland has not evolved to the same extent in the Republic of Ireland.

Irish maternity services are highly medicalised, and based almost exclusively in hospitals [5]. Busy hospital environments cannot always provide optimal supportive environments for women [6]. Ireland has limited choice in models of care of maternity care for women little progress has been made to provide alternatives to the dominant hospital-based model of maternity care [5]. Increasing economic pressures have resulted in many health services in Ireland being scrutinised with an increasing drive toward centralisation of health services. One of the two recently developed midwife-led units has been threatened with closure on the grounds of scarce resources [7], despite evidence from a randomised controlled trial demonstrating that they are actually cost-effective [8], with overwhelmingly positive birth experiences reported [9]. However, Ireland’s recently published first National Maternity Strategy recommends that women be offered choice regarding their preferred pathway of care and that all care pathways should support the normalisation of pregnancy and birth [10]

Although there is some demand for choice a medical model of care prevails, emphasising physical risk factors [5]. Women’s childbirth experiences are often obscured by invoking research based on statistical evidence and/or satisfaction studies. This research study sought to address the existing gap in knowledge about women’s preferences for experiences of childbirth in the Republic of Ireland.

## Design and methods

### Aim

To elicit women’s preferences for the most important elements of their childbirth experience.

A sequential mixed methods design was used. An initial qualitative phase consisting of 10 focus group interviews (FGIs) was followed by a Discrete Choice Experiment (DCE) to establish women’s preferences for their childbirth experiences. A more detailed account of the mixed methods design process has been published elsewhere [11]. This paper focuses on the second quantitative phase of the research design, the DCE.

### The Discrete Choice experiment

**Fig. 1:**
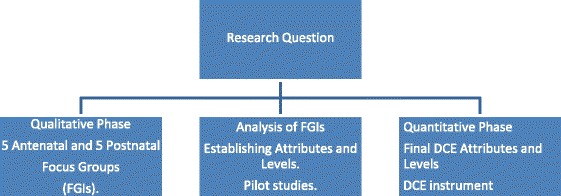
The process of developing attributes for the Discrete Choice Experiment

The DCE is a health economics tool, which relies on quantitative techniques to elicit individual preferences in relation to products or services or, in this instance, childbirth experiences. The DCE is being used increasingly in a variety of international contexts, policy developments and diverse health settings. It has been used in the elicitation of preferences for intrapartum care [12], models of labour management [13], and aspects of labour ward care [14]. The distinct stages of DCE development include identifying characteristics or attributes of the phenomena being researched, developing attributes and levels, and arranging them into scenarios or choice sets (Fig. 1).

The DCE was developed over a 12-month period and was informed by five antenatal and five postnatal focus group interviews (FGIs) with women conducted in four randomly selected and one pilot hospital in Ireland. The FGI findings have been reported elsewhere [6]. In brief, a list of six key elements or attributes of childbirth experiences were identified from the FGIs i.e. pain relief, involvement in decision-making, presence of a consultant, partnership with a midwife, interventions and individualised care (Table 1).

**Table 1:** Final attributes and levels for the DCE

Attributes	Levels
Individualised care	
Care is routine Care is individual and personal to me	Level 0 Level 1
Availability of pain relief	
I can have non-medical pain relief only I can have all types of pain relief but no epidural I can have all types of pain relief at all times but I may have to wait 3 h for epidural I can have all types available at all times	Level 0 Level 1 Level 2 Level 3
Working in partnership with the midwife	
The midwife does not work in partnership with me The midwife works in partnership with me	Level 0 Level 1
Interventions	
I get on in labour with no routine interventions It does not matter how many routine interventions I have	Level 0 Level 1
Involvement in decision-making	
Staff go ahead and make decisions for me Staff make decisions but keep me informed Staff discuss things with me before coming to a decision I am in control of decisions	Level 0 Level 1 Level 2 Level 3
Presence of consultant	
A midwife is with me during labour and birth and the consultant present only if needed such as in an emergency A midwife will be with me during labour and the consultant joins the midwife for the birth only	Level 0 Level 1

The next step of DCE development is to arrange the attributes into levels depicting a variety of experiences that are appropriate, realistic and meaningful, to that particular context [15]. The attributes and levels were then presented in a menu of hypothetical ‘scenarios’ or choice sets (Table 2). Women were then asked to select one scenario instead of another. By choosing one scenario over another, women are giving up some attributes or degrees of an attribute in order to maintain others. In economic terms this is referred to as maximising utility [15]. The DCE can therefore be used to identify not only the importance but the relative importance of attributes for women’s birth experiences.

**Table 2:** Example of a choice set with two scenarios

Scenario A ↓	Scenario B ↓
Care is routine	Care is individual and personal to me
I get on in labour with no routine interventions (like having my waters broken)	It doesn’t matter how many routine interventions I have (like having my waters broken)
I am in control of decisions	Staff go ahead and make decisions for me
I can have all types of pain relief at all times	I can have all types of pain relief (including gas and air and pethidine) but no epidural
A midwife is with me during my labour. The consultant is only present if needed, such as in an emergency	A midwife is with me during my labour. The consultant joins the midwife for the birth only
The midwife works in partnership with me	The midwife does not work in partnership with me
□	□

Tick the choice that you would prefer

Please make any comments you would like about any of the choice you were asked to make

The attribute ‘levels’ can indicate more or less of an attribute, or their presence/absence. For some attributes only two levels were identified e.g. ‘care is individual or care is routine’. For others such as ‘involvement in decision-making’ four different levels were identified from 0 (no involvement) through levels 1, 2, to level 3 (I am in control of decisions) with the respondent taking increasing responsibility for decision-making.

A pilot study of the modified DCE instrument was tested with a group of 20 women. Part of the piloting process consisted of ‘think aloud interviews’ with women to ensure they understood and agreed with all the attributes and levels. For example, women thought that waiting for up to 3 h for epidural analgesia would be acceptable; however a longer wait would affect their birth experiences (Table 1). The attribute ‘Interventions’ could be ambiguous therefore an example of an intervention was given within the attribute (“like having your waters broken”) and explained on the DCE instrument. Adjustments were made to the layout, wording, and order of questions and a further pilot study was conducted with 48 participants. Once the attributes and levels were completed they were arranged into eight choice sets with two scenarios in each. Each scenario offered a unique combination of attributes and level (Table 2). Women were invited to choose their preferred option (A or B).

The first section of the DCE instrument consisted of demographic questions, including the type of care women experienced and whether they were ‘happy’ with their birth experiences The next section included eight different choice sets (Table 2), and finally a space for women to write any additional comments about their childbirth experiences. The qualitative data from the open questions were interpreted iteratively using descriptive thematic analysis. Each response was transcribed verbatim and scrutinised for relevant extracts relating to childbirth experiences. Similarities and patterns were collated into descriptive sub themes. The sub themes were then organised into broader descriptive themes.

### Recruitment and sampling

A purposive sample of women who met the inclusion criteria, i.e., (over 18, had experienced labour, had a healthy baby, and willing to participate) were recruited through the postnatal wards of four randomly selected and one pilot hospital in the Republic of Ireland. Hospitals were stratified by annual birth rate into four groups. Birth rates < 1500, birth rates between 1500 and 3000, birth rates between 3000 and 6000, and a fourth group of hospitals with an annual birth rate over 6000. One hospital from each group was randomly selected by pulling names from an opaque envelope. Women who had given birth by elective caesarean section were excluded from the study as they had not experienced labour. However women who had experienced an initial labour and a subsequent emergency caesarean section were included.

Ethical approval was gained from four health boards and from the School of Nursing and Midwifery Research Ethics Committee, Trinity College Dublin. Consent was implied by return of a response. Midwives providing care recruited women, ensuring that they would not feel pressurised into participating. All women who met the inclusion criteria were given information and invited to participate. Those who were willing to take part were sent the DCE 6–8 weeks after birth. Each DCE was allocated a unique number to trace the response so that a reminder could be sent two weeks later. A ‘Freepost’ envelope was included, with the researchers’ contact details. Following six months of recruitment 905 participants had provided their contact details.

### Data analysis

Demographic data were analysed descriptively (SPSS version 14) using frequencies and cross tabulations. Statistical tests of significance such as chi-square tests were used for ordinal and categorical variables. Statistical significance was taken to be at least at the 0.05 level of probability.

Logistic regression appropriate to DCE analysis was used to identify the utility an individual obtains from a given combination of the birth experience attribute levels. In the context of the DCE, the ranking of the scenarios was the dependent variable and the attribute levels are the independent variables. The analysis estimated regression coefficients to identify:

- which attributes were considered important to the birth experience. If the coefficient of the attribute was found to be significant at the 5% level then it could be assumed with relative certainty (95%) that respondents considered it to be important.
- the relative importance of each attribute. The size of the coefficient made it possible to determine the importance of one attribute relative to another.

DCE data were coded and analysed with STATA (http://www.stata.com/) to calculate a ‘main effects’ model appropriate to DCE analysis. A main effects model examines the effect of one variable on another ignoring the effects of all other variables [15]. Interaction models were used to test differences in preferences in relation to hospital site, parity and education. Hospital sites were categorised according to their annual birth rates, parity as either multigravida or primigravida, and levels of education as outlined in Table 3.

**Table 3:** Demographic Characteristics of the Sample

Demographic characteristics		Values	
Age years (S.D.)	32.35	(7.1)	
Baby weeks: (S.D.)	18.0	(6.4)	
Parity N (%)	Primigravida	296	(55.7)
	Multigravida	233	(43.9)
Marital Status N (%)	Married	403	(75.9)
	Single	34	(6.4)
	Living with partner	90	(16.9)
	Other	2	(0.4)
Education N (%)	No Qualifications	17	(3.2)
	Junior Cert^a^	35	(6.6)
	Leaving Cert^b^	90	(16.9)
	Certificate/Diploma	173	(32.6)
	Degree	33	(25.0)
	Postgraduate	81	(15.3)
Ethnic background		N	%
White Irish		451	84
Other white background		56	11
'Other’		24	6

*S.D*. standard deviation

^a^Junior Certificate an intermediate examination following 3 years in secondary school (high school)

^b^Leaving Certificate examination when leaving secondary school A/O level standard or high school certificate

## Results

Five hundred and thirty-seven women returned the DCE giving a response rate of 59.3%. Six women had not completed the DCE scenarios, explaining that they ‘could not choose’ or ‘mixture of both’ scenarios offered.. These responses were excluded from analyses giving a total of 531 usable responses.

In Ireland, choice of model of care is limited; however, the FGIs identified that the type of care (private or public) was an important component of women’s experiences of childbirth. ‘Domino care’ refers to a type of public community care available to women in certain catchment areas. (See Appendix). Therefore, in addition to the demographic data, women were asked about the type of care they accessed. Due to constraints of word count, four of the variables are reported here:

- type of care (public, private, or semiprivate)
- type of birth (vaginal, instrumental or caesarean section)
- pain relief used.
- happiness with experiences

### Type of care and type of birth

The demographic characteristics of the sample are outlined in Table 3. Most women had given birth 12–24 weeks prior to completing the DCE. Table 4 shows that 283 women (53.2%) availed of public maternity care and 246 women (46.3%) availed of private or semiprivate care. Three women availed of ‘DOMINO’ or community care. They are included in the ‘public care’ statistics. The Overall normal vaginal birth rate was 66% (Table 4). The rate of normal births for women using the public maternity services was 72.4% (*n* = 205) while for women attending private or semiprivate care the rate was 58% (*n* = 143). The caesarean section rate for women using the public service was 8.8% (*n* = 25), while women accessing private or semiprivate care the rate was more than double at 20.3% (*n* = 50) (Table 4). The rates of instrumental births were also slightly higher in the private and semiprivate groups than in the public group. A statistically significant relationship was noted between type of care and type of birth (χ^2^ = 17.1 df = 3, *p* < .001).

**Table 4:** Type of care, type of birth and pain relief used

Type of care	N (%)	Vaginal birth	Instrumental birth	Emergency caesarean section	No pain relief	Non medical pain relief	Pethidine/Entonox	Epidural analgesia	Mixture of both
Public	283 (53.2)	205 (72.4)	53 (18.7)	25 (8.8)	43 (15.1)	12 (4.2)	105 (37.1)	72 (25.4%)	54 19.0
Private/semiprivate	246 (46.3)	143 (58)	53 (21.6)	50 (20.3)	32 (13)	5 (2)	65 (26.4)	105 (42.6)	39 (15.9)
Total^a^	531	351 (66)	106 (19.9)	75 (14.1)	75 (14.1)	17 (3.2)	170 (32)	177 (33.3)	93 (17.5)

^a^Values from two respondents missing

### Pain relief

Epidural analgesia was the most frequently used method of pain relief (33.3%, *n* = 177) followed by pethidine and Entonox at 32% (*n* = 170) (Table 4). In addition 17.5% (*n* = 93) of respondents used a mixture of pain relief measures. Non pharmacological pain relief was used by just 3.2% (*n* = 17), and 75 women (14%) did not use any pain relief. Epidural analgesia was used by 25.4% (*n* = 72) of public care users compared with a higher percentage 42.6% (*n* = 105) of private and semi-private care users. A chi-square test indicated that there is a statistically significant association between the type of care and type of pain relief used. (χ^2^ = 18.504. df = 4 *p* < .001).

### Happy with their experience

Most women described themselves as being ‘happy’ or ‘very happy’ with their birth experience (84.1%, *n* = 447). When the type of birth was cross tabulated with women being happy with their experience of birth, the group who were happiest were women who had a normal vaginal birth. Three hundred and eleven women (89.3%) who experienced a normal birth described themselves as being ‘very happy’ or’ very happy’. Women who experienced a caesarean section, (*n* = 75,) 53 women (70.7%) reported being ‘happy’, or ‘very happy’, with their birth experiences, whilst 12 women (16%) said they were either ‘unhappy’, or ‘not at all happy’. There was a statistical relationship between the type of birth and women’s feelings of happiness with the birth experience (χ^2^ = 22.394. df = 4 *p* < 0.0005) (Table 5).

**Table 5:** Combined type of birth and happy with experience

Type of birth		Not sure	Happy/very happy	Not happy/not at all happy	Total
Normal vaginal birth	N (%)	17 (4.9)	311 (89.3)	20 (5.7)	348^a^
Caesarean section		10 (13.3)	53 (70.7)	12 (16)	75

^a^Values missing

### Discrete choice experiment

When participants chose one scenario over another in each of the eight choice sets, the DCE analysis identified which attributes were most important to women. Based on the direction (positive or negative) and significance of the regression coefficients, four of the six attributes were found to have a significant influence on women’s preferences for their childbirth experiences. Individualised care, pain relief at all levels, consultant presence, and partnership with the midwife are all significant and hence are important to women’s birth experience. Interventions and decision-making at levels 1, 2, and 3, are non-significant (Table 6).

**Table 6:** Logistic Regression showing significance of attributes and levels

Attribute	Coefficient (95% CI)	*P* value^a^
I can have all types of pain relief but no epidural	0.897	<0.001
I can have all types of pain relief but may have to wait 3 h for epidural	0.872	< 0.001
I can have all types of pain relief at all times	1.620	< 0.001
The midwife works in partnership with me	0.995	< 0.001
Staff make decisions but keep me informed	0.061	0.334
Staff discuss with me before coming to a decision	0.043	*0.546*
I am in control of decisions	−0.073	*0.249*
A midwife is with me during my labour. The consultant joins the midwife for the birth only	−0.116	*0.00*2

^a^ *p* values <0.05 are statistically significant

### Interpretation of the results

A positive coefficient means that the level 1 (2 or 3) option is preferred to the level ‘O’ option. A negative coefficient means that the ‘0’ level is the preferred option of the two. Only if the *p* value is less than 0.05 can the difference be considered statistically significant (Table 6).

**Table 7:** Thematic analysis of open questions

Themes	Frequency
1. Relationship with staff	78
2. Pain relief	58
3. Support	38
4. Description of childbirth	43
5. Environment	29
6. Interventions	24
7. Expectations/information	14
8. Comments about DCE	7
Total	291

Women identified choice of pain relief at all levels as the most significant attribute. Relatively speaking therefore, women demonstrated a preference for scenarios that offered increased availability of pain relief. Having access to all types of pain relief all the time had the greatest influence on which scenario women chose, hence this was the most important attribute identified by respondents.

Partnership with the midwife was the second most important attribute of the birth experience and was statistically significant. Women valued this attribute highly, and chose scenarios where they were more likely to work in partnership with the midwife.

Although the attribute ‘interventions’ has a positive coefficient, it did not reach statistical significance. The numbers of interventions were therefore not a significant preference for birth experiences for most women.

The attribute of decision-making at all levels was found to be non-significant and would therefore not be considered as relatively less important as other preferences for respondent’s birth experiences.

The ‘presence of the consultant’ results showed a negative coefficient indicating that women’s preference was to have a consultant present in an emergency only, rather than have a consultant present at the birth.

An interaction model was conducted to test the impact of parity, education, or hospital site on preferences for pain relief. The results showed no significant difference within these variables. Therefore across all strata the choice of pain relief was considered an important preference for women’s childbirth experiences.

### Integration of data from open questions

A total of 291 respondents (54.9%) made comments, which ranged from a sentence to a page in length. Table 7 shows the thematic analysis and the number of comments relating to each of the eight themes. The discussion here is restricted to two key themes: relationship with carers, and pain relief. Some choice sets involved ‘giving up’ elements that may have been important to women and they often illuminated their choice of scenario with an explanation.

For example, one respondent explained the rationale for her choosing pain relief within each of the eight choice sets as follows;

- Choice 1: ‘all types of pain relief is important because you don’t know how it’s going to go on the day’;
- Choice 2: ‘Again pain relief is why I picked B’;
- Choice 3: ‘What makes B attractive is all types of pain relief but also being in control, the midwife working with me, and being in control of decisions’;
- Choice 7: ‘Again the pain relief swung it for me but it is a disgrace to have to wait for an epidural’;
- Choice 8: ‘Again the pain relief is the most important; people should always be given the option of having an epidural’.

### Relationship with midwife

Comments related to the attribute ‘partnership with the midwife’ suggested that certain characteristics of the midwife enhanced or detracted from the relationship. There were 151 comments about aspects of care by midwives. They describe the midwife as “brilliant”, “wonderful”, “calm,” “natural”, “experienced”, “compassionate”, “gave excellent care”. “She anticipated my needs” and ‘engaged with me”. Negative comments included midwives being “harsh”, “unsympathetic”, and “irritated by my crying”, “talking over me,” “nervous, consulting others all the time”. Women seemed to be happy with midwifery care and one suggested that the “consultant [was] not needed”.

### Pain relief

There were 94 comments relating to women’s accessibility to pain relief, their rationale and context for the choice of pain relief and its effectiveness. Women felt that they would be considered “daft not to have pain relief”. A third of women mentioned that pethidine and entonox were “useless” or “didn’t work”. The most frequently mentioned pain relief method was epidural analgesia, generating 53 comments. Two-thirds of comments were positive “would recommend it totally,” “helped me feel comfortable,” “relaxed,” “in control”, “such relief”. Ten women referred to having non-pharmacological or no pain relief. One woman commented that there was a lack of information about non-pharmacological pain relief, and complementary therapies “were not encouraged” whereas pharmacological pain relief was “pushed at classes”.

Women having their first baby frequently commented they had been advised or “warned to have epidural” by friends; they were fearful about not being able to access epidural analgesia, stating that they “feel happier with [the] possibility of epidural”, or being “terrified not to have [the] epidural”. Women expressed delight when they have achieved a ‘normal labour’ but: “with the help of the epidural”. Several women expressed that an epidural was essential if they wanted to give birth ‘normally’. One woman who had already given birth commented “epidural is the only way I would have another.” Despite the dependence on pain relief and a reluctance to rely on their own coping mechanisms most women appeared to construe labour as ‘normal’ unless they gave birth by caesarean section.

Seventy-five women (14%) had no pain relief and commented that they were “proud”, or “glad to have done without epidural”. Some women expressed appreciation about not being forced to have an epidural noting that their “birth plan was respected,” and that they “felt better with no epidural,” and were “glad [to be] allowed get on without [an] epidural.” Few women were totally committed to the ‘natural’ belief system, though, and tended not to have confidence in their own abilities without the help of modern technology. “I would never have done it without the epidural”, some found it difficult to understand those who rejected relief from pain. One suggestion that rejection of pain relief is old fashioned “The modern way is to get rid of the pain”…“why suffer?”

## Discussion

The DCE established that women set clear priorities, preferring all types of pain relief to be available to them at all times, individualised care, midwives working in partnership with them, and the presence of a consultant for emergencies only. Decision-making during labour and the use of interventions were not significant elements of the childbirth experience when women were confronted with scenario choices. There was little impact on women’s choices when the interactions of hospital site, parity, and education were considered. ‘Pain relief’ was a strong influencing factor in women’s choices with increased availability of pain relief options increasing the likelihood that women would express their preferences for those options. The findings of the regression model suggest that the second most important attribute for women’s preferences was ‘working in partnership with the midwife’ followed by ‘individualised care’ and that the least important attribute was ‘decision-making’.

Similar to previous work, this study indicates a high acceptability of the DCE for women using maternity services [13, 14]. The qualitative and quantitative elements of the design augmented the findings of the DCE so that although women’s priorities for childbirth experiences were identified statistically, information from the open questions provided richer contextual perspectives. For example, it was evident that women wanted a blend of attributes personalised to their own individualised requirements that were contingent on the environment and the support they received. This is understandable, given the stressed maternity hospital environment in Ireland, where staff have little time to support women in labour [11]. Interventions were taken as part and parcel of the childbirth process, not disapproved of, and often welcomed. Women appeared to trust that any interventions would be done in their best interest. Women did not express a need to be involved in decision-making in relation to interventions, and did not appear to associate epidural analgesia with any negative effects on their labour.

A recent study comparing women’s choices of pain relief in midwife-led and consultant-led units in Ireland showed that when women were offered other options such as hydrotherapy and transcutaneous electrical nerve stimulation, fewer women chose epidural analgesia [9]. Women in the present study expressed intense emotions such as “horror” and “terror” at the thought of not being able to access pain relief. Although women thought the relationship with the midwife was important, pain relief often ‘trumped’ that choice. It has been suggested that women who access an epidural feel they can be less reliant on the support of a midwife [16]. In a medicalised system, where women have little opportunity for choice and control, it could be hypothesised that the availability of an epidural is one way in which women can perceive that they have some control over childbirth [17].

In this study, few women expressed the possibility of using their own coping strategies or non-pharmacological methods of pain relief. There appeared to be little encouragement for women to develop or investigate alternatives both antenatally and during labour and birth. Hospital environments, which have been described as hierarchical and unsupportive to midwives attempting to normalise childbirth [18] may have inhibited midwives in helping them.

Many women believed that having a ‘contingency plan’ was eminently sensible and viewed childbirth with uncertainty. Women in this study did not want to be committed to either dichotomy of ‘natural’ or ‘medical’ typologies, or to be caught in the middle between competing ideologies [19]. Although women wanted birth experiences that were ‘as natural as possible,’ they did not want to be in a position where they were locked into that decision and unable to change their minds; thus the prevailing dichotomy of ‘natural’ versus ‘medicalised’ care that forms the basis of much of the maternity care literature [20] is not an acceptable one for women.

Most women expressed a ‘wing it and see’ attitude, having technology as a ‘safety net’ to be used if required. Women’s flexibility could be a compromise of ideals, where women lower their expectations as a ‘survival strategy’ to lessen the dissonance between their anticipation and their actual experiences [2]. This suggests that the polarised debates engaged in by health professionals, of ‘medicalised’ versus ‘natural’ may not always be helpful to women in their care.

In addition to valuing technology, women rated having a relationship with the midwife highly but second to the availability of pain relief. Although a systematic review exploring factors influencing women’s evaluations of their childbirth experiences, found that attitudes and behaviours of caregivers were more powerful influences than pain relief and intrapartum interventions [21] it was not the case with this study. Women articulated a medicalised perspective of childbirth, where they felt they often needed help and were unable to ‘go it alone’. Preferences could vary in a different setting such as midwifery led care or home births. The prioritising of pain relief can therefore be interpreted in this particular context. Although an antipathy to childbirth pain was often expressed, epidurals seemed to be a panacea for other uncertainties and gave an element of choice, control, and a feeling of relief. Maternity care provision in Ireland is primarily based in hospital. This may suggest that availability of pain relief increases the demand, or that offering pain relief to women in labour is irresistible [22]. It was evident from this study that many women viewed epidural analgesia as ‘normal’. Women were sometimes disappointed not to have experienced an epidural because they were too advanced in labour.

Although the presence of a consultant has been shown to be valued highly by women in Ireland [6], in the context of other attributes it was deemed by this sample of women to be not as important, although appreciated in an emergency. Availing of private, consultant-led care resulted in a higher caesarean section rate (20.3%) than for those women attending the public service (8.8%), similar to findings from Brazil [23] and Australia [24].

Decision-making was similarly found to be less important for women in the context of having other attributes such as individualised care, possibly because of the ‘entangled’ nature of decision-making in childbirth [25]. Similar to van Teijlingen et al’s [19] findings, most women who had not experienced a less medicalised system thought that what they received must be ‘the best’; therefore, in their minds, there was no competing ideology between ‘medicalised’ and ‘natural’ birth. Although recent evidence suggests that alternative models of midwifery care are safe and cost-effective, and should be an option for all women [26], hospital based, consultant-led medicalised care continues to be the norm for women in Ireland.

### Limitations

The limitations of the study are the relatively homogenous group that responded to both phases of the study. ‘Discrete choices’ were difficult to construct due to the qualitative nature of women’s preferences. Attributes identified were specific to the context of maternity care in Ireland within a medicalised environment where private maternity care is seen as desirable by many women and cannot, necessarily, be generalised to all of the population. The response rate is also a little low (59.3%), although in keeping with other studies in this field.

## Conclusions

Women’s childbirth experiences are located in a particular cultural and political environment, and it is clear that individuals and groups of people involved in maternity care construe childbirth experiences differently. Priorities for childbirth experiences are couched in cultural and contextual factors. Service users who have no experience of non-medicalised maternity care continue to value the current model of care provision. This study suggests that polarised debates, dichotomized services and competing ideologies do not empower women. An understanding by health professionals that, rather than concentrate on either ‘medicalised’ or ‘natural’ birth, focussing on individualising care and normalising each woman’s experience will ultimately provide the high quality care that labouring women require.

Alternatives such as midwife-led care and community midwifery services with transparent and supportive interfaces with other models of care will need further development to provide more balanced choices for women. The ultimate goal is for women in Ireland to have optimum birth experiences in an environment where they feel safe and supported. This study has shown that it is possible to move beyond binary indices to evaluate experiences. Measuring and identifying attributes using both quantitative and qualitative means determines the components of childbirth experiences that are important to women and provides more contextual understanding. Women need to be supported to have more confidence in their own capabilities. More research, particularly from a national perspective, is required to develop maternity services that respond to women’s needs. There is a particular necessity to focus on birth experience and its potential to empower women at this important time in their lives.

## Appendix

Types of maternity care available to respondents.

Public maternity care is free for all women. It includes all antenatal care, care during labour and childbirth, and postnatal care. A midwife cares for the woman during labour and birth referring to a doctor if there are any deviations from the norm or in the case of an emergency. It covers all hospital accommodation costs. Women are cared for by midwives in the postnatal period in hospital. Women visit their General Practitioner for a postnatal check-up.

Private maternity care, is where a woman has an identified consultant obstetrician who is paid a fee; usually through a private insurance system. Women attend the consultant antenatally, some consultants will be available for the birth. After the birth women can avail of a private room if it is available. Women usually visit the consultant for a postnatal check-up.

Semi-private care the configuration of care varies between in hospitals. Women are seen by their own consultant but the doctor that is on duty usually attends the birth. Some hospitals provide ‘semi-private’ clinics and women can accessed semiprivate facilities.

Domino Care refers to domiciliary ‘In’ and ‘Out’ care. Women within certain catchment areas can avail of community midwifery care. Women are usually seen antenatally by a team of community midwives in combination with their General Practitioner. Birth takes place ‘In’ the hospital and the woman has the choice of early transfer home 6–12 h later where she is visited by the community midwives in the postnatal period up to ten days depending on the model being used.
